# Boolean factor graph model for biological systems: the yeast cell-cycle network

**DOI:** 10.1186/s12859-021-04361-8

**Published:** 2021-09-17

**Authors:** Stephen Kotiang, Ali Eslami

**Affiliations:** Department of Electrical Engineering and Computer Science, Wichita State University, Wichita, KS 67260 USA

**Keywords:** Boolean networks, Factor graph, Network perturbation, Systems biology

## Abstract

**Background:**

The desire to understand genomic functions and the behavior of complex gene regulatory networks has recently been a major research focus in systems biology. As a result, a plethora of computational and modeling tools have been proposed to identify and infer interactions among biological entities. Here, we consider the general question of the effect of perturbation on the global dynamical network behavior as well as error propagation in biological networks to incite research pertaining to intervention strategies.

**Results:**

This paper introduces a computational framework that combines the formulation of Boolean networks and factor graphs to explore the global dynamical features of biological systems. A message-passing algorithm is proposed for this formalism to evolve network states as messages in the graph. In addition, the mathematical formulation allows us to describe the dynamics and behavior of error propagation in gene regulatory networks by conducting a density evolution (DE) analysis. The model is applied to assess the network state progression and the impact of gene deletion in the budding yeast cell cycle. Simulation results show that our model predictions match published experimental data. Also, our findings reveal that the sample yeast cell-cycle network is not only robust but also consistent with real high-throughput expression data. Finally, our DE analysis serves as a tool to find the optimal values of network parameters for resilience against perturbations, especially in the inference of genetic graphs.

**Conclusion:**

Our computational framework provides a useful graphical model and analytical tools to study biological networks. It can be a powerful tool to predict the consequences of gene deletions before conducting wet bench experiments because it proves to be a quick route to predicting biologically relevant dynamic properties without tunable kinetic parameters.

**Supplementary Information:**

The online version contains supplementary material available at 10.1186/s12859-021-04361-8.

## Background

In biological networks, the temporal evolution of gene or protein expressions constitutes a dynamical system. Modeling the coupled dynamics and characterization of the long-run behavior of such networks is perhaps the most important task in genomic signal processing. In the literature, long-run distribution has been conjectured to correspond to the phenotype of a cell [1]. Consequently, different analytic and computational models have been proposed to capture the behavior of complex gene regulatory networks, including differential equations [2, 3], Bayesian networks [4], and Boolean networks (BNs) [5, 6]. Among deterministic dynamical systems, perhaps the BN model has received the most significant research effort since it was introduced by Kauffman [1, 7]. BNs constitute an important class of models for regulatory networks of gene interactions, in that they are simple and capture some fundamental characteristics of gene regulations, and their rule-based structure carries physical and biological meaningful phenomena, for instance, stability, hysteresis, cellular state dynamics, and the possession of a switch-like behavior [8].

In this paper, our goal is to predict the impact on the long-run behavior and network state progression caused by perturbation (also referred to as disturbance) of regulatory functions. In addition, we provide an exact analytic characterization of error evolution in biological networks, in particular, errors due to state disturbances on nodes. In systems biology, we can think of errors as a result of noise emanating from either environmental or biological fluctuations that influence a biological process [9, 10]. For example, “genetic switches” that control cellular decisions in gene networks can alter the pattern of gene expression under a small change in external stimuli such as mutagens, pH changes, heat stress, etc. Also, genetic switches can flip, especially when random fluctuations bring the system close to the threshold for a transition [9]. In addition, fluctuations can propagate to a higher level of biological organization and affect biological functions such as decision-making, spatiotemporal population dynamics, and even evolutionary processes. Here, our study focuses on BNs with perturbation, particularly with a focus on gene deletions and random state perturbation. Note that allowing genes to randomly flip states is biologically meaningful [9, 11].

In the literature, the dynamical properties of Boolean networks have been studied based on two fundamental types of perturbations: state and structural. In state perturbation, genes or protein expression states in the network are flipped to modulate the dynamics. State perturbation is considered temporary because it resets the initial states of the underlying deterministic rule and does not alter the network structure [11]. Hence, the network attractors and the basins of attraction remain invariant. However, if the BN model has multiple attractors, state perturbations may cause convergence to a different attractor than the original one and may lead to a change in the steady-state distribution of the BN. State perturbations have been studied mostly by analyzing the collective behavior of a large number of random BNs [11–13]. On the other hand, structural perturbation, also referred to as functional perturbation [14, 15], has a more fundamental impact on BNs. The long-run distribution is changed permanently, or the progression of states is halted since the underlying rule-based structure is altered. As a result, functional perturbation has the potential to reverse or force the gene network to transition from undesirable stable states, which is a useful tool in developing gene therapies. Functional perturbations are less studied, and most algorithms proposed are rather cumbersome because they require the computation of transition probabilities before and after perturbations. Also, most perturbation studies employ Markov chain [16] analysis to empirically estimate the steady-state distribution of a network. For the first time, we introduce the “density evolution” (DE) analysis [17] to study state perturbations in gene networks, hoping this could potentially be employed in rigorous analysis for functional perturbations.

In the literature, several methods have been proposed to qualitatively reproduce some known dynamical features of the wild-type biological systems, as well as the consequences of single gene deletions. Based on the differential equation model in [18], the authors used numerical integration techniques to model the control of the restriction point of the mammalian cell cycle. However, this model appears difficult to extend. Also, Fauré et al. [19] applied a logical modeling technique to delineate the main dynamical properties of the mammalian cell cycle network. They assessed the merits and limits of synchronous network updating assumptions in BNs against asynchronous assumptions. However, in their model, the effect of each regulator depends on the presence of co-regulators. Similarly, many simulation and analysis software tools for logical models exist, including GINsim [20], BoolNet [21], bioLQM [22], and CellNetAnalyzer [23]. However, these tools rarely consider error analysis in biological networks.

Other tools of dynamical systems theory like bifurcation analysis [24] and time-scale analysis based on the sign of Jacobian eigenvalues [25] provide temporal patterns that are often comparable to experimental data, which is a real advantage. Moreover, DNA content analysis by flow cytometry [26, 27] has been employed to study the effects of single gene deletion and gene over-expression on cell cycle progression. Such models contain detailed information about time evolution of the system. However, modeling the actual time duration of cellular processes requires knowledge of a large number of biochemical parameters that are difficult to find [28]. In addition, when interest is in the prediction of the sequential pattern of states and the long-run distribution of cellular processes, the exact time course of the regulatory network dynamics may be neglected. A recent report indicates that some gene networks are so robustly designed that timing is insignificant [29].

In this work, we propose a computational framework that combines the formulation of BNs [1] and factor graphs [30, 31] to investigate the global dynamical property and impact of gene knockout in regulatory networks of gene interactions. With the flexibility and genericity of factor graph formalism [30], we believe that the methods proposed here will aid in the analysis of Boolean genetic graphs using a wide range of biological rules or processes. We formalize the model as a Boolean factor graph and propose a message-passing protocol to evolve network states. The framework and structure of our proposed model can allow us to track the progress of network states. Thus, it has the potential for supporting network intervention analysis. We employed a synchronous updating scheme in our model. The synchronous update approach is chosen for simplicity; however, in reality, molecular processes or events are not coordinated in time. Also, in gene knockout analysis, the requirement for the accurate specification of time delays or of priorities that are difficult to define or may be context-sensitive obscures the implementation of an asynchronous update strategy.

Furthermore, the proposed framework allows us to derive an analytic closed-form recursive equation that captures the behavior and propagation of errors introduced by random state perturbations in gene networks through a density evolution analysis. Here, we applied our methodology to study a sample network regulating the cell cycle of the budding yeast, referred to as the Li model [32]. In [32], the authors did not systematically analyze the effect of reported gene deletions. Our simulation results on yeast cell-cycle gene deletion analyses are supported by experimental data in the literature. In addition, our findings show that the Li model is consistent with real gene-expression data. From our analyses, we deduced that error characterization is not only important in its own right but also forms a basis that allows us to quantify network parameters in designing models for inferring gene regulatory networks from gene-expression profiles. Finally, the application of our derived recursive equation elucidates what properties of gene networks are directly responsible for their robustness.

## Model and methods

This section provides a background on Boolean networks as a model for representing gene regulatory networks, and factor graphs, which are required to understand the proposed model and the analysis in this paper. We then introduce and describe a message-passing protocol employed on the proposed model to evolve network states as messages. Finally, we present a brief introduction of a sample biological network used for application of our model and methods.

### Boolean networks

Formalism of the BN model underscores the fundamental generic principles rather than quantitative biochemical details, which establishes a natural framework for capturing the dynamics of gene networks and their regulatory mechanisms, yielding insights into their overall behavior. For consistency of notation with materials in the literature [5], we define a Boolean network G(V,F) by a set of *n* binary-valued nodes $V = \{x_{1}, \ldots , x_{n} \}$ and a list of Boolean functions $F = \{f_{1}, \ldots , f_{n} \}$. In systems biology, the set of nodes *V* could represent biological entities such as genes, mRNAs, and transcription factors (TFs). Each node $x_{i} \in \{0,1\}$ has $k_{i}$ parent nodes (i.e., regulators). Also, we denote $Pa_{i} = \{Pa_{i,1}, Pa_{i,2}, \ldots , Pa_{i,k_{i}} \}$ as the set of parents of $x_{i}$. For clarity, in this work, we refer to the biological entities as genes of a network. The state of $x_{i}$ denotes the expression of the node quantized to only two levels. In this model, $x_{i} = 1$ means that gene *i* is expressed (active), and $x_{i} = 0$ means that it is not expressed (inactive). Whenever a gene is expressed, it could affect the expression or suppression of other genes. Therefore, the value or state of a gene at time $t+1$ is given deterministically by its regulators at time *t* through a Boolean function $f_{i} \in F$ as

1 $$\begin{aligned} x_{i}(t + 1) = f_{i}\left( x_{i1}(t), x_{i2}(t), \ldots , x_{ik_{i}}(t) \right) \,, \end{aligned}$$

where $\{i1, \ldots , ik_{i}\} \subseteq \{1,\ldots , n\}$, and $k_{i}$ is the connectivity of node $x_{i}$. The network function *F* represents the rules of regulatory interactions between the genes. Given the network state at time *t* as $\mathbf{x} (t) = \left( x_{1}(t), \ldots , x_{n}(t)\right)$, the state transition $\mathbf{x} (t) \rightarrow \mathbf{x} (t+1)$ is governed by *F* as $\mathbf{x} (t+1) = F\left( \mathbf{x} (t)\right)$. In addition, if $Pa_{i} = \emptyset$ (i.e., $x_{i}$ has no parents), then $x_{i}(t+1) = x_{i}(t)$.

**Fig. 1:**
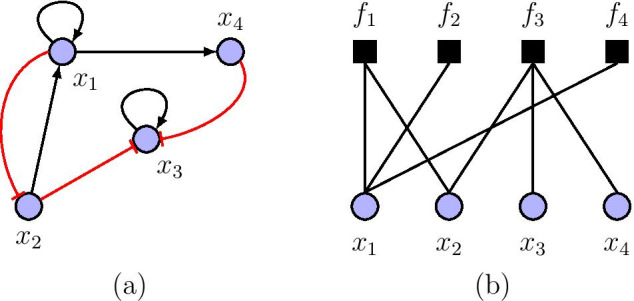
**a** Simple directed gene graph with $n=4$. **b** Equivalent undirected factor graph representation of (**a**). The state of gene $x_{i}$ at time $t+1$ is governed by Boolean function $f_{i}$, given a set of interacting genes. For instance, $x_{3}(t+1)= f_{3}(x_{2}(t), x_{3}(t), x_{4}(t))$. Red blunt edges indicate inhibition links, whereas black arrowheads represent activation interactions

The set of all possible states (i.e., state space) of the BN contains $2^{n}$ network states, so that after a finite number of state transitions, the initial sequence becomes transformed into a stable sequence of zero-dimensional fixed points known as singleton attractors. In certain instances, the initial sequence may eventually transition into a set of cyclical attractors. All states that lie on trajectories flowing to an attractor comprise its basin of attraction. Of note, the attractors capture the long-run behavior of a dynamical system. Also, in biological systems, the key idea is to perceive each stable attractor configuration as representing one possible biological phenotype or cell type [1]. An example of a simple Boolean genetic graph with four genes is shown in Fig. 1a. In this network, a directed arrow edge implies an activation interaction link, while a blunt edge denotes an inhibition influence.

Furthermore, we presume that any node out of the *n* possible nodes can get perturbed independently of other nodes. In the BN setting, network state perturbation is represented by a random flip of value from 0 to 1, or vice versa. Since the genome is an open system with external inputs, it is known that genes may become either inhibited or activated due to external stimuli. In our model, an error is introduced into the network with a positive probability $\epsilon \ll 1$ when the state of a node changes due to random gene perturbations. (See further analysis in the density evolution section).

### Boolean factor graph model

In biological systems, for each cell type and for each function performed by a cell, the regulatory network has a specific form that determines what biochemical processes will be executed and in what order. For instance, in Li’s yeast cell-cycle model, the stationary G$_{1}$ attractor configuration has 13 state transitions once the cell has committed to division (see Table 2 in [32] as well as Additional file 1: Table S1). Therefore, attractors bear strong biological implications, and the question of interest is to understand their nature and properties, and how they respond to perturbation in the network. In this paper, we study and analyze the behavior of gene networks by representing them in terms of Boolean factor graphs.

A factor graph, also referred to as a bipartite graph, associates variable nodes (symbolized by circles in Fig. 1b) on one side of the graph and control nodes (symbolized by squares in Fig. 1b) on the other side [30]. Factor graphs subsume many graphical models in probability theory, signal processing, and coding, including Markov random fields [33], Bayesian networks [34], and Tanner graphs [17, 35]. It is plausible that many algorithms and mathematical models in these fields are naturally expressed in terms of factor graphs. One such algorithm is the $\mathsf {sum\text {-}product~ algorithm}$ [30], which operates in a factor graph by passing “messages” along edges of the graph, following a single, simple computational rule, as a decoding algorithm for low-density parity-check codes [17]. In a quest to study error correction in biological systems, authors in [36] established a direct relationship between BN models of gene regulatory networks and bipartite graphs used in decoding algorithms in coding theory [35]. This relationship stems from a key experimental observation in that biological networks have sparsely distributed and possibly long edges [1]. See Additional file 1 on introduction to factor graphs.

In this context, a variable node denotes a gene $x_{i}$, whereas a control node represents a Boolean function, $f_{i}$. Figure 1b shows the equivalent bipartite form of the Boolean gene graph in Fig. 1a. To convert a Boolean network to a bipartite graph, we simply draw an edge between a variable node $x_{i}$ and a control node $f_{j}$, if the scope of $f_{j}$ contains $x_{i}$. In simple terms, a gene connected to a control node $f_{i}$ exerts an influence on the operation of gene $x_{i}$ within the assumption of one time unit. For instance, in Fig. 1b, genes $x_{2}$ and $x_{4}$ exert an influence on gene $x_{3}$ through the Boolean function $f_{3}$ following the deterministic Eq. (1). The factor graph representation is convenient and has been utilized widely in the literature but in a probabilistic setup [30, 37, 38]. In addition to the structure of our proposed model, in this section, we describe a simplified Boolean function model at the control nodes and formulate a message-passing algorithm as an inference tool to evolve network states.

#### Boolean functions

Boolean functions consist of a set of rules specifying how a given node in a graph changes its value over time, as a function of the past or current states of its parent nodes. These functions represent the simple dynamics of inhibition and activation between interacting nodes. In [6], Martin et al. used an activation-inhibition Boolean function model as an inference algorithm to reverse engineer the regulatory network of gene interactions from microarray time series data. As an example, in this work, we model a simplified Boolean function that takes into consideration the current state of the regulated node $x_{i}$. We hope that this model, though simple, may still preserve certain biologically meaningful patterns of interactions. A similar Boolean model was employed in [19, 20], where the logical combination of interactions on a regulated node was compared to the concentration/activity level of that node to make a decision on the new concentration level.

By definition, we formulated the Boolean function $f_{i}$ at the control node for a variable $x_{i}$ using activation and inhibition functions depicted by the truth tables shown in Table 1.

**Table 1:** Boolean truth tables for both activating and inhibiting gene interactions

Activation			Inhibition		
$x_{1}$	$x_{2}$	$x_{2}^{\prime }$	$x_{1}$	$x_{2}$	$x_{2}^{\prime }$
0	0	0	0	0	0
0	1	1	0	1	1
1	0	1	1	0	0
1	1	1	1	1	0

Column $x_{1}$ represents the state of a regulator node, and column $x_{2}$ denotes the child node state at time *t*. The output state of the child node at time $t+1$ is denoted by column $x_{2}^{\prime }$. For activation, $x_{2}^{\prime } = x_{1} \vee x_{2}$, and for inhibition, $x_{2}^{\prime } = (x_{1} \oplus x_{2}) \wedge x_{2}$. Only when a parent node is active does it contribute information to the child node

Our activation-inhibition Boolean functions take into account the present state of the child (i.e., regulated) node. In accordance with the logical rule in [32], our Boolean functions stipulate that only when a regulator node is active does it contribute information to the child node. For instance, given two interacting nodes in a network where $x_{1}$ activates $x_{2}$, the state of node $x_{2}$ at time $t+1$ is defined by a Boolean function as $x_{2}(t+1) = x_{1}(t) \vee x_{2}(t)$. Similarly, if $x_{1}$ inhibits $x_{2}$, then $x_{2}(t+1) = \left( x_{1}(t) \oplus x_{2}(t)\right) \wedge x_{2}(t)$. The logical operators $\{ \vee , \wedge , \oplus \}$ used bear the usual meanings, and all operations are in GF(2).

In this work, we have considered simple deterministic rules for illustration of the proposed model. However, due to the simple nature of factor graphs, many other typical processes of biological systems or complex rules may easily be emulated. Such processes may include cooperative effects of active regulators, targeted inhibitions, longer activation times for certain nodes, etc. For instance, we can implement logical cooperative effects at a control node following the activation-inhibition function proposed in [6] of the form $x(t+1) = \left( x_{a1}(t) \vee x_{a2}(t) \vee \cdots \right) ~ \wedge \lnot ~\left( x_{r1}(t) \vee x_{r2}(t) \vee \cdots \right)$, where $x_{a1}, x_{a2}, \ldots$ are activators, and $x_{r1}, x_{r2}, \ldots$ are inhibitors or repressors acting on a node. The operator $\lnot$ denotes a logical NOT. We can implement such activation-inhibition functions as a single conceptual computational rule.

#### Message-passing algorithm for network inference

Having formulated the Boolean functions at the control nodes, here, we develop and describe a message-passing algorithm as an inference technique to evolve network states as messages on the Boolean factor graph. Message-passing techniques such as junction tree, sum-product, and belief propagation have been successfully employed in the decoding of codes on graphs [17, 30, 34]. Similarly, since gene regulatory networks are cyclic in nature, a variant of the message-passing algorithm referred to as loopy belief propagation has been employed as an inference tool in biological systems, albeit in a probabilistic setting [37, 39].

**Fig. 2:**
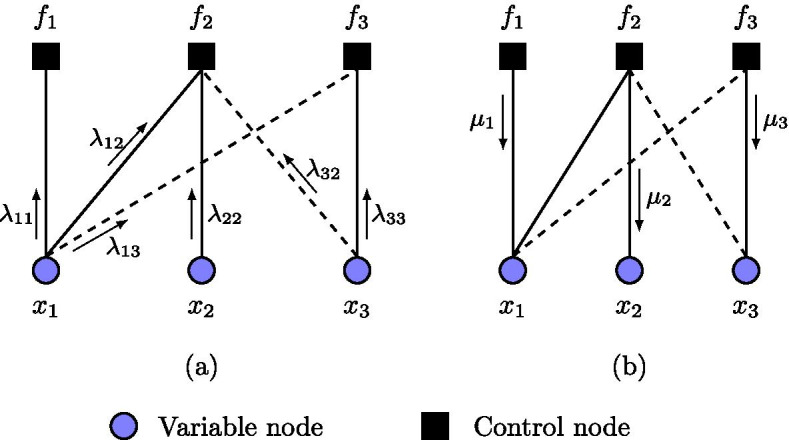
Message passing update in a sample Boolean factor graph: **a** messages sent from variable nodes to control nodes denoted by $\lambda$ values, and **b** messages sent from control nodes to variable nodes given by $\mu$ values. Solid (dashed) edges denote activation (inhibition) interactions

Here, the evolution of network states begins at the variable nodes of a factor graph. At the beginning, we initialize the variable nodes of the factor graph with one of the possible $2^{n}$ network states. Each variable node performs no computation, but simply sends out its current state as a message to all its neighboring control nodes, including its corresponding control node. Formally, we denote a message sent from a variable node, *i*, to a control node, *j*, as $\lambda _{ij}$, where $\{i, j\} \subseteq \{1,\ldots , n\}$, as shown in Fig. 2a.

Therefore, a control node $f_{i}$ receives a set of $k_{i}$ messages from its neighboring variable nodes, in addition to message $\lambda _{ii}$. Recall that $k_{i}$ is the cardinality of regulators of gene $x_{i}$. For each message $\lambda _{ij}$ received at $f_{i}$, $f_{i}$ computes a value (0 or 1) of what the next state of $x_{i}$ should be, based on $\lambda _{ij}$ and $\lambda _{ii}$ using the Boolean function truth tables in Table 1. Then, $f_{i}$ performs majority voting among these $k_{i}$ values to form a belief $\mu _{i}$ as the next state of $x_{i}$, as shown in Fig. 2b, and sends it to $x_{i}$. We perform the majority rule to adapt our Boolean functions in accordance with logical rules in [32]. In the next iteration or time step, $x_{i}$ sends out its acquired new state. For example, consider control node $f_{2}$. In each iteration, $f_{2}$ receives messages $\lambda _{12}$, $\lambda _{22}$, and $\lambda _{32}$. Using the activation Boolean function truth table, $f_{2}$ computes an output value using $\lambda _{12}$ and $\lambda _{22}$ since node $x_{1}$ activates node $x_{2}$. Similarly, since node $x_{3}$ inhibits node $x_{2}$, $f_{2}$ uses the inhibition Boolean function truth table to compute an output value based on incident messages $\lambda _{22}$ and $\lambda _{32}$. Then, $f_{2}$ performs a majority voting over all output values to form a belief $\mu _{2}$ and sends this value to node $x_{2}$ as its new state.

In our proposed model, we assume that at each iteration, all nodes are synchronously updated in accordance with the regulatory rules assigned to them, and this process is then repeated. The network is said to have attained a stable sequence if, at time *t*, the value of variable nodes are invariant for all times $t^{\prime } \ge t$.

### Model network: yeast cell cycle

**Fig. 3:**
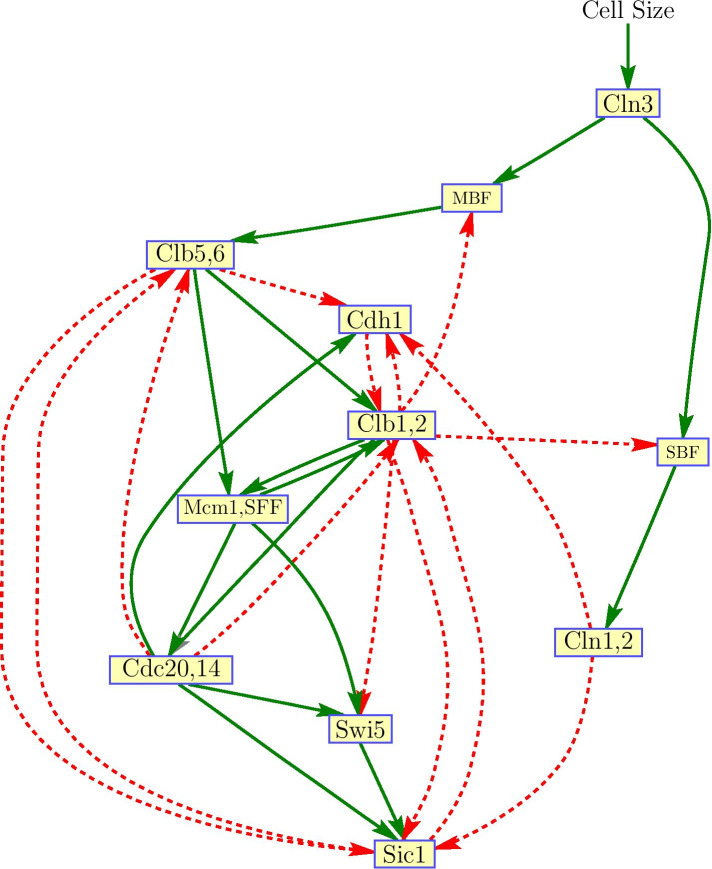
Simplified yeast cell-cycle network adapted from [32]. Solid green edges denote activation links, whereas dashed red edges represent inhibition links. Nodes Cln3, Cln1,2, Swi5, Cdc20,14, and Mcm1,SFF have self degradation. Cln3 is a “stater kinase” used to trigger the G$_{1}$ - S phase transition when the cell grows sufficiently large

In this paper, the yeast cell-cycle network model presented by Li et al. [32] is used as an illustrative example to demonstrate the application of our proposed model and methodologies in systems biology. This network was constructed using experimentally verified and known key regulators reported in the literature. Figure 3 shows the connectivity among the various nodes with corresponding interaction type.

The logical network consists of 11 nodes participating in the regulatory process that controls the cell cycle in budding yeast. This process consists of four phases: G$_{1}$, S, G$_{2}$, and M. At the G$_{1}$ phase, the cell grows and commits to division under appropriate conditions. In the S phase, DNA is synthesized and chromosomes are replicated. G$_{2}$ is a “gap” between S and M. The final phase, M, corresponds to mitosis, in which chromosomes are separated and the cell divides before returning to the G$_{1}$ phase, thereby completing one cycle. The M phase encompasses several subphases, namely prophase, metaphase, anaphase, and telophase. In the model, nodes are classified into four classes: cyclins (G$_{1}$ cyclins Cln1,2 and Cln3, and the S/M cyclins Clb1,2 and Clb5,6), inhibitors of cyclin/Cdk1 complexes (Sic1, Cdh1, Cdc20,14), transcription factors (MBF, SBF, Swi5, and Mcm1,SFF), and the checkpoint cell size. Studies on cell cycle rely mainly on cell size changes to initiate cell division at a point called “Start” in budding yeast [26, 40]. For example, when a yeast cell evaluates its growth in the late G$_{1}$ phase and moves to the S phase, it commits itself to a new round of DNA synthesis and mitosis before returning to G$_{1}$.

Though not depicted in Fig. 3, nodes Cln3, Cln1,2, Swi5, Cdc20,14, and Mcm1,SFF have self-degradation. In [32], the authors implemented self-degradation as a time-delayed interaction at the variable nodes. That is, if the state of a self-degrading node at time *t* is 1 and for the entire delay period $t_{d}$ the states of its regulator nodes are 0, then at time $t + t_{d}$, the node will be degraded to 0. In our simulations, we set $t_{d} = 1$. According to [32], under a synchronous network-update scheme, the logical network in Fig. 3 has seven singleton attractors or fixed points, with the largest attractor consisting of 1,764 states ($\approx 86\%$ of the total state space, i.e., $2^{11}$) as its basin size. This large fixed point is consistent with the stationary G$_{1}$ phase of the cell cycle, in which the cyclin/Cdk1 inhibitors Sic1 and Cdh1 are expressed while all other nodes are inactive [3]. This is referred to as the G$_{1}$ attractor. Of the seven attractors, only the G$_{1}$ attractor represents an observable biological state, because under normal conditions, the cell will be sitting in this state unless perturbed. Moreover, Li and colleagues performed network perturbation by deleting or adding an interaction edge, as well as changing the activation and inhibition links. They observed that for most perturbations, the relative changes of the basin size of the G1 attractor were small. In summary, Li et al. [32] concluded that this yeast cell-cycle logical network is robustly designed.

## Applications of Boolean factor graph model

In this section, we demonstrate the performance of our proposed model by providing three use case examples, namely gene-deletion analysis, network consistency analysis, and node connectivity analysis. We deduce biological insights based on the findings.

### Gene-deletion analysis

Gene deletion, also widely known as gene knockout (KO), is a type of perturbation on the network structure. This structural perturbation alters the connectivity or Boolean functions of the network and, as a result, may lead to changes in the functionality of a biological network. When structural changes occur, the network fixed points and basins of attraction will be impacted and subsequently its long-run behavior. These changes can be permanent unless an intervention is implemented. A salient motivation for studying structural perturbation include the following: (1) biological systems are modular, robust, and subject to uncertainties; thus, it is desirable to elucidate the effect of a small difference in network models on their dynamic behavior; (2) gene regulations have intrinsic stochasticity, and it is of interest to predict the outcome of any change in regulation; and (3) it is important for practical use, such as design and analysis of therapeutic intervention strategies [14]. Also, gene KO analysis could lead to a knowledge of critical nodes in a network whose perturbation leads to significant functional changes in the biological system in order to reduce the network size by eliminating the redundant components.

In this section, we employ our Boolean factor graph model to verify the impact of gene KOs on the yeast cell-cycle progression based on the Li model. We note that in biological gene KO experiments, the expression of a target protein or gene molecule is stopped by eliminating the protein-coding regions from the genome. Therefore, in our case, we modified our factor graph model accordingly by fixing the state of the target node to zero and eliminating the corresponding control node. We account for the viability of a budding yeast cell cycle if it is able to go through all four phases (G$_{1}$/S/G$_{2}$/M) having 13 state transitions (see Additional file 1: Table S1). Subsequently, to validate our model, we compared our model simulation findings to the published experimental observations on gene KO experiments. Our simulations confirm biological results in budding yeast cell-cycle experiments. We deduce that our model can possibly be used in predictive gene KO analysis.

#### Deletion of G$_{1}$ stabilizers

Deletion of all G$_{1}$ stabilizers (Sic1 and Cdh1) results in inviable cells [3]. This lethality might be caused by deletion of Sic1, which creates some DNA damage checkpoint (not modeled here) that would arrest the cells in the telophase, M phase. Furthermore, deletion of either Cdh1 or Sic1 allows the cell to undergo a start. In our model, Cdh1 deletion results in a viable cell, which is consistent with the literature [41]. However, in Sic1 KO, though the mutant cell is able to replicate its DNA, it gets stuck in the telophase, as reported by [3, 42]. Also, the authors in [43] studied the degradation of mitotic cyclins in sic1 deletion yeast strains, reporting that degradation of the cyclin subunit requires inhibition of the mitotic kinase-mediated by Sic1. They further observed that sic1 deletion mutant strains were inviable. Table 2 presents our model simulation results of the evolution of protein states for Sic1 KO, indicating that the cell-cycle sequence goes from the excited state and then arrests in the M phase.

**Table 2:** Temporal evolution of protein states in Sic1 gene deletion

Time	Genes											Phase
	Cln3	MBF	SBF	Cln1,2	Cdh1	Swi5	Cdc20,14	Clb5,6	Sic1	Clb1,2	Mcm1,SFF	
1	1	0	0	0	1	0	0	0	**0**	0	0	Start
2	0	1	1	0	1	0	0	0	**0**	0	0	G$_{1}$
3	0	1	1	1	1	0	0	1	**0**	0	0	S
4	0	1	1	1	0	0	0	1	**0**	0	1	G$_{2}$
5	0	1	1	1	0	1	1	1	**0**	1	1	G$_{2}$
6	0	0	0	1	0	1	1	1	**0**	1	1	M
7	0	0	0	0	0	1	1	0	**0**	1	1	M

The cell cycle gets stuck in the M phase. The “Start” phase refers to a series of linked events that prepare a cell for budding and DNA replication. Completion of the “Start” phase and commitment to a new cycle of cell division precedes the actual end of the G$_{1}$ phase. Bold states in the sequence rows denote the state of the deleted node. Also, the number of time steps in each phase does not reflect its actual duration

According to a study by Hoose et al. [26], any gene deletion that changes the length of the G$_{1}$ phase relative to other cell-cycle phases will alter the DNA content profile. In yeast, DNA content analyses have been used to measure the effects of cell-cycle arrest when essential genes are either knocked out [27] or over-expressed [44]. In their work, Hoose et al. [26] reported that the majority of gene deletions affecting cell progression lead to a lengthened G$_{1}$ phase. However, they also observed that cells lacking Sic1 (Cdk inhibitor of Clb/Cdk complexes) move more quickly into the S phase. That is, the mutant cell goes through a shorter G$_{1}$ phase, representing premature DNA replication and genome instability [42]. Applying our model confirms this, as we observed that Sic1 gene deletion results in only one time step of the G$_{1}$ phase, compared to three time steps in a normal cell (see Table 2 in [32] as well as Additional file 1: Table S1). Furthermore, in the literature, it has been reported that Cdc20 transcription is activated in the M phase by a transcription factor complex Mcm1/SFF, which is activated in turn by Clb1,2 [3]. Thus, the activation of gene Cdc20 drives the cell progression from the M to G$_{1}$ phase. According to our model simulation result, deletion of Cdc20,14 blocks cells in the M phase (Additional file 1: Table S2), which is again consistent with other published reports [3].

#### Deletion of G$_{1}$, S, and M cyclins

Using our model, we first knocked out both Cln3 and Cln1,2, and observed that the cell fails to execute “Start” and remain in the stationary G$_1$ phase, as shown in Table 3.

**Table 3:** Yeast cell cycle progression in Cln3 and Cln1,2 deletion

Time	Genes											Phase
	Cln3	MBF	SBF	Cln1,2	Cdh1	Swi5	Cdc20,14	Clb5,6	Sic1	Clb1,2	Mcm1,SFF	
1	**0**	0	0	**0**	1	0	0	0	1	0	0	Stationary G$_{1}$

Bold states in the sequence rows denote the state of the deleted node

Accordingly, in the literature, deletion of all three Cln genes arrests cells in the G$_{1}$ phase because the start-signal facilitators are missing, and the cell is not able to bud [3]. Clb1,2 is essential for successful mitosis. The lack of Clb1,2 is lethal as the cell arrests in the G$_{2}$ phase, because other Cdk/cyclin complexes cannot initiate mitosis [41, 42]. This lethality underscores the key role of Clb1,2 in regulating cell-cycle events. Table 4 shows our model’s temporal evolution of protein states in a Clb1,2 gene-deletion simulation.

**Table 4:** Temporal evolution of protein states in Clb1,2 gene deletion

Time	Genes											Phase
	Cln3	MBF	SBF	Cln1,2	Cdh1	Swi5	Cdc20,14	Clb5,6	Sic1	Clb1,2	Mcm1,SFF	
1	1	0	0	0	1	0	0	0	1	**0**	0	Start
2	0	1	1	0	1	0	0	0	1	**0**	0	G$_{1}$
3	0	1	1	1	1	0	0	0	1	**0**	0	G$_{1}$
4	0	1	1	1	0	0	0	0	0	**0**	0	G$_{1}$
5	0	1	1	1	0	0	0	1	0	**0**	0	S
6	0	1	1	1	0	0	0	1	0	**0**	1	S
7	0	1	1	1	0	1	1	1	0	**0**	1	G$_{2}$

The cell cycle arrests in the G$_{2}$ phase. Bold states in the sequence rows denote the state of the deleted node

Here, too, we see that our Boolean factor graph model is able to confirm the impact of single gene knockouts consistent with the literature. Furthermore, from the literature, it is known that Clb5,6 is responsible for the initiation of DNA replication in the S phase [27, 41]. In our model, the absence of Clb5,6 stops cell progression into the S phase, as expected (see Additional file 1: Table S3). As observed, protein evolution arrests on the fourth time step of Table 4. The cell cannot initiate DNA synthesis and exhibits a G$_{1}$ arrest phenotype.

#### Deletion of transcription factors

In the Li model, when the yeast cell size reaches a threshold, Cln3 activates SBF and MBF, the transcription factors of Cln1,2 and Clb5,6, respectively. Although the Clb5,6 level rises, it is inhibited by the G$_{1}$ stabilizer, Sic1. However, since Cln1,2 cannot be repressed by Sic1, it can phosphorylate Sic1, making it susceptible to degradation. Consequently, Clb5,6 becomes active and phosphorylates the second G$_{1}$ stabilizer, Cdh1, resulting in complete Cdh1 inactivation.

In applying our model, we first deleted SBF and MBF and observed that the suppression of both of these transcription factors causes the cell to arrest before the “Start” transition, which is consistent with published materials [40]. Table 5 shows our model’s temporal evolution in this case.

**Table 5:** Temporal evolution of protein states in SBF and MBF TFs deletion

Time	Genes											Phase
	Cln3	MBF	SBF	Cln1,2	Cdh1	Swi5	Cdc20,14	Clb5,6	Sic1	Clb1,2	Mcm1,SFF	
1	1	**0**	**0**	0	1	0	0	0	1	0	0	Start
2	0	**0**	**0**	0	1	0	0	0	1	0	0	Stationary G$_{1}$

Bold states in the sequence rows denote the state of the deleted node

The cell cycle arrests before the “Start” transition (i.e., cells do not progress, even into the first G$_{1}$ phase)

Conversely, the absence of either SBF or MBF is sufficient for budding yeast cells to execute “Start,” as was also observed in our simulation (Additional file 1: Tables S4 and S5). However, the cell arrests in the G$_{1}$ phase. This observation confirms published experiment reports about the two TFs. Of note, SBF is composed of two components, Swi4 and Swi6 genes, and inhibited by Whi5, whereas MBF is composed of Swi6 and Mbp1 genes [45, 46]. Consequently, Kraikivski et al. [40] reported that a mutant yeast cell with a single-gene deletion of either Swi4 or Mbp1 is viable.

### Gene network consistency analysis

Another application of our proposed model is to test the consistency of the existing biological networks against real gene-expression data, that is, to quantify how well a network is supported by data. As an example, we considered the Li’s model yeast cell-cycle network. It is our understanding that this kind of consistency analysis on the yeast model has not been carried out in the literature. We obtained a dataset of uniformly normalized expression profiles from the M$^{3\mathrm {D}}$ database [47]. This compendium data provides a bulk download of human-curated, computable experimental metadata and computer-validated data for integrity. Compendium data used on the yeast genome (version 3 build 2) contain 904 microarray profiles collected under a wide range of experimental conditions, including wild-type, gene(s) deletion, varying oxygen concentrations, fermentation, sporulation, different media, etc. For our analysis, we first discretized the gene-expression data.

#### Discretization of data

In the literature, several methods have been proposed to discretize or cluster gene-expression data [39, 48]. In general, discretization is carried out if prior biological knowledge suggests that the underlying variables are indeed discrete, or for computational efficiency. Furthermore, since discretized data can be more stable with respect to random variations of gene-expression measurements [48], discretization can help improve the robustness of data and reduce noise in the continuous variables. According to Gat-Viks et al. [39], the variable-specific discretization method outperforms the global optimized single common discretization scheme. In addition, it is generally more accurate and flexible than standard clustering preprocessing methods used in [4] for real gene-expression data. However, this flexibility may come at a cost of over-fitting and decreased learnability.

In this section, we employed a gene-specific discretization scheme that optimizes the Gaussian mixture model likelihood using the iterative expectation-maximization (EM) algorithm in the MATLAB environment. In each EM iteration, we infer the posterior distributions of component memberships and use these to re-estimate the mixture proportions by computing the Gaussian sufficient statistics (component means, covariance matrices, and mixing proportions). The new discretization distributions are used in the next iteration, and the algorithm iterates until convergence.

Here, we used $\mathsf {fitgmdist}$ function in MATLAB to model the relations between the continuous observations on a gene and its discrete logical state. $\mathsf {fitgmdist}$ implements the iterative EM learning algorithm to fit a mixture of Gaussian models to data. By default, $\mathsf {fitgmdist}$ implements the $\mathsf {k-means}++$ algorithm for initialization to choose ${\mathsf {k}}$ initial cluster centers. In this paper, a Gaussian component corresponds to a specific logical state of a gene. Here, we used ${\mathsf {k}} = 2$. Also, we note that each node’s state may designate a different range of gene-expression levels defined by the estimated parameters (i.e., mixture proportions, mean, and variance statistics) of the Gaussian mixture model on each node.

For each discretization of gene data, we repeated the EM algorithm ten times using a new set of initial cluster values and a maximum number of 1,000 iterations allowed. Then, we computed the Bayes information criterion (BIC) score of our discretization model to quantify how good the gene expression fits with the mixture of two Gaussian models. A likelihood-based measure of model fit to compare multiple models fit to the same data is BIC = $2*\mathsf {NlogL} + {\mathsf {p}}*\mathsf {log(n)}$, where $\mathsf {NlogL}$ is the negative loglikelihood, ${\mathsf {n}}$ is the number of observations, and ${\mathsf {p}}$ is the number of estimated parameters specified as a numeric vector of length ${\mathsf {k}}$. The model with the lowest BIC score is the best fitting model. Table 6 shows BIC measures of our gene-specific discretization for ${\mathsf {k}}=1, \ldots , 5$. Except for Cdh1, Clb5,6, and Clb1,2 gene-expression data that have ${\mathsf {k}}=3$ as the best model fit, the remainder of the genes have ${\mathsf {k}}=2$ as the optimal mixtures of Gaussian model distribution. Therefore, we used only ${\mathsf {k}}=2$ logical states $\{0,1\}$ for all nodes, corresponding to the up-regulation (1) and down-regulation (0) of genes, in conformity with Boolean models.

**Table 6:** Bayes information criterion (BIC) measure of Gaussian mixture model discretization used to fit gene-expression data for different ${\mathsf {k}}$ number of components

Genes	BIC Scores ($1\times 10^{3}$)				
	${\mathsf {k}}=1$	${\mathsf {k}}=2$	${\mathsf {k}}=3$	${\mathsf {k}}=4$	${\mathsf {k}}=5$
Cln3	2.6183	**2**.**6020**	2.6193	2.6397	2.6600
MBF	2.0492	**1**.**8838**	1.8913	1.9039	1.9182
SBF	2.0083	**1**.**9608**	1.9640	1.9781	1.9901
Cln1,2	2.6117	**2**.**3853**	2.3923	2.4117	2.4318
Cdh1	1.7429	1.4212	**1**.**4066**	1.4234	1.4404
Swi5	2.3690	**2**.**1339**	2.1354	2.1468	2.1660
Cdc20,14	1.7202	**1**.**6175**	1.6211	1.6326	1.6525
Clb5,6	2.0188	1.9799	**1**.**9333**	1.9374	1.9499
Sic1	1.4909	**1**.**4785**	1.4824	1.5027	1.5232
Clb1,2	2.5741	2.5267	**2**.**5218**	2.5330	2.5470
Mcm1,SFF	1.5210	**1**.**4607**	1.4757	1.4957	1.5123

The lowest BIC value is the best fitting model, as highlighted in bold

#### Results

First, we verified that our proposed methodology accurately reproduced the attractors distribution as reported in [32], with the largest fixed point attracting $86\%$ of the 2048 initial states. Then, we employed our proposed Boolean factor graph model to study the state evolution of the discretized gene-expression data. From the data, we have a total of 904 initial states. That is, each experimental observation point equals a state sequence in the 11-node logical network. Starting from each of the 904 initial states, we find that all of these states eventually flow into one of the two fixed points shown in Table 7.

**Table 7:** Attractors of cell cycle on real biological data

Basin size	Genes										
	Cln3	MBF	SBF	Cln1,2	Cdh1	Swi5	Cdc20,14	Clb5,6	Sic1	Clb1,2	Mcm1,SFF
822	0	0	0	0	1	0	0	0	1	0	0
82	0	1	0	0	1	0	0	0	1	0	0

Each stable point is represented in a row. The genes’ columns show the state of a gene in the respective fixed point

Remarkably, using real biological data, the G$_{1}$ attractor is the largest fixed point, attracting 822 ($\approx 90.9\%$) of the 904 initial states. Additionally, we implemented a similar discretization scheme as above; however, we used random sampling to select ${\mathsf {k}}$ initial cluster centers. Under this discretization scheme, we observed that the initial states eventually flow into the two fixed points shown in Table 7 with the same G$_{1}$ attractor, attracting $91.92\%$ of states. As we expected, the percentage of states in the G$_{1}$ attractor using real gene-expression data is comparably and above the $86\%$ wild-type basin size. Based on these results, we can deduce that even under diverse experimental conditions, the stability of the cell state is guaranteed. Thus, we can consider the basin of attraction of the G$_{1}$ attractor as the allowable states that the cell can assume under external influence or stimuli. Once the stimuli are removed, the cell flows back to the stationary state. In addition, these findings may imply that the Li model is consistent with the microarray data obtained from various biological experiments. Of note, if there was a disparity between the data and the network considered, then we expected to see more initial states transitioning to other attractors than to the G$_{1}$ attractor.

### Node connectivity analysis

In this section, we apply our proposed Boolean factor graph model to study the impact of node connectivity in biological networks and analytically characterize the dynamics of error propagation and recovery in Boolean gene networks. We assume that an initial random state perturbation introduces an error in the Boolean network. A state perturbation may result from either environmental or biological fluctuations that affect cellular decisions in gene networks. For our analysis, we consider random networks with given degree distributions as models of genetic graphs. This enables us to capture biological networks with arbitrary degree distributions. Of note, the degree of a node in a factor graph is the number of edges incident to it. For an ensemble of random Boolean networks, we use polynomials to represent the degree distributions of the networks as

2 $$\begin{aligned} \rho (x) = \sum _{j \ge 1} \rho _{j} x^{j} \,, \end{aligned}$$

where $\rho _{j}$ denotes the fraction of edges incident to a control node with degree *j*, constrained to

3 $$\begin{aligned} \sum _{j \ge 1} \rho _{j} = 1 . \end{aligned}$$

**Fig. 4:**
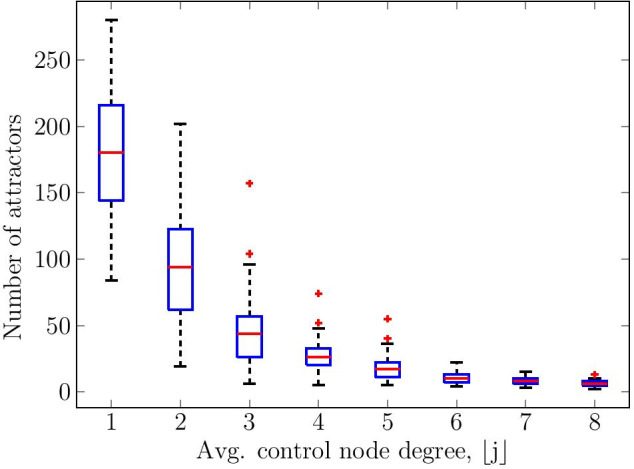
Boxplots of the number of attractors of random biological networks with increasing average connectivity. Each network has ten nodes with irregular control node degree distribution, and $\lfloor j \rfloor$ denotes the largest integer that is less than or equal to *j*. The median of the boxplots follows a power-law distribution

To demonstrate how node connectivity would influence the stability of a biological network, we studied an ensemble of random networks with ten nodes. For each experiment, we sampled at least 50 networks with control nodes having irregular degree distributions to mimic real biological networks, and we set $\lfloor j \rfloor \in \{1, 2, \ldots , 8 \}$ to denote the average connectivity of the network. Moreover, each network has random types of edge influence (activation and inhibition). Starting from each of the $2^{10}$ initial states, we allowed the states to evolve and then counted the number of resultant fixed points. Figure 4 shows boxplots of the resultant number of network attractors with increasing average control node degree, $\lfloor j \rfloor$. We observed that the number of attractors, as depicted by the median of the boxplots, has a power-law distribution. Based on this observation, we can gain useful insights into the nature of biological graphs. With increasing average node degree *j*, the basin size of the stationary attractor increases. Consequently, the homeostatic stability of a cell increases monotonically. Furthermore, for the ten-node random networks considered, we observed that as *j* becomes greater than 6, there are instances of both singleton and cycle attractors. This is interesting since in the literature it has been reported that large-scale or highly interconnected networks converge into a complex attractor where the system irregularly oscillates among a set of states, especially when an asynchronous update scheme is employed [49]. We deduce that in biological Boolean networks, nodes with a higher degree of connections are likely the key contributors to the presence of attractor cycles. Similarly, biological networks where $\rho (x) = x$ are basically unstable, and any error caused by a random disturbance on a node cannot be corrected unless the node is self-regulating.

#### Density evolution

To capture the impact of random perturbations in BNs, we borrow the concept of density evolution used in the performance analysis of factor graph models [17, 50]. The performance of such graphical models depends on the degree distributions of their nodes on the graph [51]. In message-passing algorithms employed on factor graphs, DE refers to tracking the evolution of the probability density function of error messages between variable nodes and control nodes. Here, for the first time, we apply DE analysis to study biological networks. We hypothesize that DE can be used to provide an exact analytic characterization of the impact on the cell attractors caused by state and/or structural perturbations. The result is a closed-form formula referred to as a “DE equation.” In this paper, we derive and employ the DE equation to provide numerical and analytical investigation of random state perturbations on the resiliency and robustness of biological networks.

For Boolean networks with perturbation, an error is introduced with a positive probability $\epsilon \ll 1$ by which the state of a node is randomly changed. Implicitly, we assume that there is an independent identically distributed (i.i.d.) random perturbation over the variable nodes in the graph. Based on our proposed Boolean factor graph model, we track the evolution of this perturbation following the message-passing protocol established in the model and methods section, for both activation and inhibition interactions. If this probability decreases at the end of each iteration, then the network will attain the G$_{1}$ attractor, whereas if this probability increases, then spurious attractors will be obtained.

**Fig. 5:**
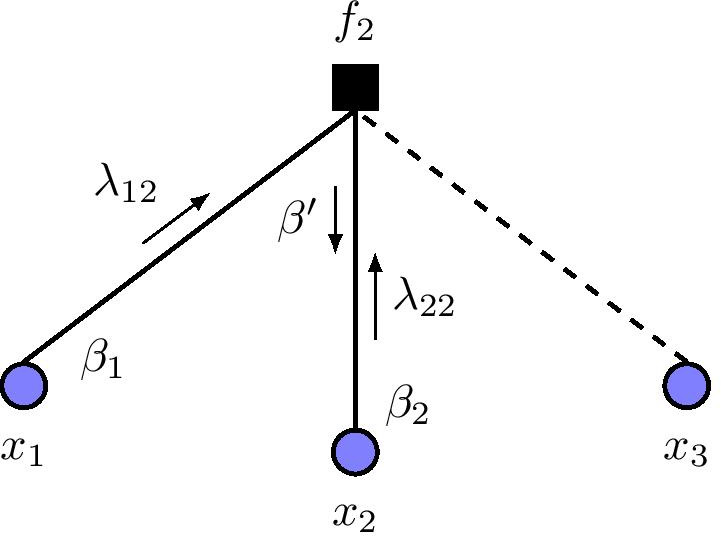
Message passing in Boolean factor graph with state perturbation probability $\epsilon$. Gene $x_{1}$ ($x_{3}$) activates (inhibits) gene $x_{2}$. $\beta$ is an event that a gene is perturbed. The dashed edge denotes inhibition interaction. Messages $\lambda$ are passed between variable nodes and control nodes

Figure 5 shows the evolution of random errors in a three-gene Boolean factor graph model.

For our analysis, we first consider the activation interaction link, whereby gene $x_{1}$ activates $x_{2}$. Let messages $\lambda _{12}$ and $\lambda _{22}$ denote the states of variable nodes $x_{1}$ and $x_{2}$ sent to the control node $f_{2}$ in the $(l-1)$-th iteration of the message-passing procedure. Also, let $\beta _{1}$ and $\beta _{2}$ be events whereby messages $\lambda _{12}$ and $\lambda _{22}$ have occurred in error, respectively, as shown in Fig. 5. In addition, $\beta ^{\prime }$ is the event that there is an error in the output message of control node $f_{2}$ to variable node $x_{2}$. We further assume that perturbations that introduce errors occur with equal probability on any variable node. Therefore, using the Boolean function truth tables in Table 1, the probability that there is an error in the *l*-th iteration of node $x_{2}$ can be described in terms of the $(l-1)$-th iteration (i.e., $\epsilon _{l} = f(\epsilon _{l-1})$) as

4 $$\begin{aligned} \begin{aligned} \epsilon _{l}&= p(\beta ^{\prime } | \beta _{1}, {\overline{\beta }}_{2}) \cdot p(\beta _{1}, {\overline{\beta }}_{2}) + p(\beta ^{\prime } | {\overline{\beta }}_{1}, \beta _{2}) \cdot p({\overline{\beta }}_{1}, \beta _{2}) \\&~~~~~~~ + ~p(\beta ^{\prime } | \beta _{1}, \beta _{2}) \cdot p(\beta _{1}, \beta _{2}). \\&= \frac{1}{2} \epsilon _{l-1}(1-\epsilon _{l-1}) + \frac{1}{2} (1-\epsilon _{l-1}) \epsilon _{l-1} + \frac{1}{2} \epsilon _{l-1}^{2}. \\&= \epsilon _{l-1} \left( 1 - \frac{1}{2}\epsilon _{l-1} \right) \,. \end{aligned} \end{aligned}$$

Similarly, by considering the inhibition edge in Fig. 5, the error probability in the *l*-th iteration of node $x_{2}$ can be obtained using the Boolean truth table for inhibition. The iterative equation for inhibition would be the same as Eq. (4). Supposing that node $f_{2}$ is of degree *j*, then at the *l*-th iteration, the total propagated error probability in the message sent from $f_{2}$ to node $x_{2}$ is given by

5 $$\begin{aligned} \epsilon _{l} = \sum _{k = \left\lceil {\frac{j-1}{2}} \right\rceil }^{j-1} {j-1 \atopwithdelims ()k} y_{l-1}^{k} \left( 1-y_{l-1}\right) ^{j-1-k} \,, \end{aligned}$$

where $y_{l-1} = \epsilon _{l-1} \left( 1 - \frac{1}{2} \epsilon _{l-1} \right)$. In our proposed model, a variable node sends its current state to its connected control nodes in the subsequent iteration. Therefore, given a biological network with random state perturbations and control node degree distribution $\rho (x)$, the average error probability on any particular gene node can be described by the recursive DE equation model as

6 $$\begin{aligned} \epsilon _{l} = \sum _{j=1}^{dc} \rho _{j} \left[ \sum _{k = \left\lceil {\frac{j-1}{2}} \right\rceil }^{j-1} {j-1 \atopwithdelims ()k} y_{l-1}^{k} \left( 1-y_{l-1}\right) ^{j-1-k} \right] \,, \end{aligned}$$

where $d_{c}$ is the maximum degree of the control nodes.

By having a DE equation at hand, various connectivity analyses can be conducted. A simple but interesting one is as follows. Consider the DE equation (6). By expanding the right-hand side of the equation, we obtain

7 $$\begin{aligned} \begin{aligned} \epsilon _{l} =&~\rho _{1} + \left( \rho _{2} + 2 \rho _{3}\right) \epsilon _{l-1} + O(\epsilon _{l-1}^{2}) \,. \end{aligned} \end{aligned}$$

For $\epsilon _{l}$ to be less than $\epsilon _{l-1}$ for every *l* (i.e., vanishing state disturbances), it is necessary that $\epsilon _{l-1}$ be larger than the first two terms on the right-hand side of Eq. (7). That is

8 $$\begin{aligned} \rho _{1} + \left( \rho _{2} + 2 \rho _{3}\right) \epsilon _{l-1} < \epsilon _{l-1} \,. \end{aligned}$$

Thus,

9 $$\begin{aligned} \rho _{2} + 2 \rho _{3}&< 1, \end{aligned}$$

10 $$\begin{aligned} \rho _{3}&< 0.5 . \end{aligned}$$

**Fig. 6:**
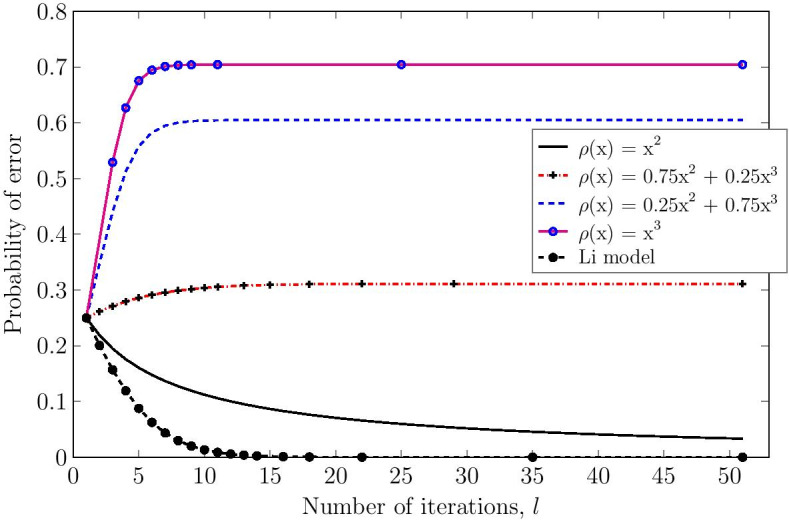
Error probability under density evolution for networks with different $\rho (x)$ as $l \rightarrow \infty$. Initial $\epsilon _{0} = 0.25$

The inequalities in Eqs. (8)–(10) provide some interesting intuitions. First, note that $\rho _{1}$ is a constant, and as such, errors on degree-one distribution nodes (i.e., genes without regulators) do not vanish unless they are self-regulating. Second, the inequalities (9) and (10) indicate that in order to achieve a resilient genetic network, a large portion of the control nodes should have degree $j > 3$. However, while *j* is in theory unbounded and can be equal to *n*, i.e., number of nodes, we note that gene networks follow a power-law distribution with an exponent greater than 2 [52]. This restricts the upper bound of *j*. In our model, control nodes with higher degrees provide more information about the true state of their corresponding variable nodes from neighboring nodes. Figure 6 shows an error evolution (initial $\epsilon _{0} = 0.25$) for a set of different control node degree distributions in genetic graphs as the number of message-passing iterations grows. Moreover, we have included the Li model with a network distribution given by $\rho (x) = 0.025x + 0.05x^{2} + 0.3x^{3} + 0.2x^{4} + 0.125x^{5} + 0.3x^{6}$ in Fig. 6. As expected, a violation of inequalities (9) and (10) is such that $\epsilon _{l} \not \rightarrow 0$. Therefore, we note that in the inference of BNs from gene-expression profiles, the DE equation can allow us to determine the degree distribution restrictions on the nodes for diminishing errors arising from random state perturbations. These observations have significant implications on models used for inferring biological Boolean graphs from gene-expression data in reference to network stability. In such models, the authors have often limited the degree of connectivity to less than or equal to 3, citing model complexity and poor performance metrics [5, 6].

## Performance analysis

### Models comparison

In this section, we provide a qualitative and quantitative comparative analysis of traditional Boolean approaches used to analyze budding yeast as reported in the literature and as given by our model’s simulation output. We compared our model to GINsim [20] and BoolNet [21] software tools that are based on logical formalism.

#### GINsim model

In [53], the authors employed the GINsim model [20] to conduct a comparative study of logical models of cell cycle control in eukaryotes. For example, the authors encoded and adapted Li’s budding yeast model [32] by transcribing the logical rules into the GINsim model. Although the global topology of the logical network is preserved, the authors introduced positive feedback loops on several nodes, namely MBF, SBF, Clb5,6, Clb1,2, Cdh1, and Sic1. In contrast, the self-degradation loops seen in Li’s model were eliminated (Figure 1, top left in [53]).

Subsequently, in the analysis of the functionality of regulatory circuits of the resultant network model, Fauré et al. [53] deduced that the positive self-activating loops help in the maintenance of alternative, artefactual stable states. Using proper logical rules and employing a synchronous update scheme, the authors observed that all trajectories in the state space of the revised network converge towards a single stable state corresponding to the G$_{1}$ attractor. Therefore, our model’s finding is consistent with the GINsim analysis of the yeast cell cycle model as reported in the literature, in particular regarding the dominant stable state of the logical network.

According to our deduction, the GINsim model does not readily allow for the implementation of a majority voting rule thus obscuring a direct comparison with our proposed model. Besides, our model can provide non-binary logical analysis by defining appropriate non-binary logical functions at the control nodes and employing a non-binary message-passing algorithm.

#### BoolNet model

Here, we employed the BoolNet model [21] to provide a comparative study of the dynamical behavior of Li’s logical network. Using the $\mathsf {BoolNet}$ package in the R environment, we transposed the logical network as a text file (see the $\mathsf {cell\_cycle.txt}$ file in the dedicated GitHub repository indicated in the “Availability of data” section) containing temporal elements and encoded it in a symbolic form, i.e., as expression trees [21]. We implemented a majority voting rule on the network nodes using the $\mathsf {maj()}$ command available in the $\mathsf {BoolNet}$ package. Also, we employed time delays to transcribe self-degradation loops in the resultant logical network. However, according to our evaluation, the BoolNet model does not take into consideration the current state of the regulated node in deciding the next state of the node. Incorporating the current state of the regulated node in the BoolNet model creates a self-regulating loop. This hindered the full implementation of our model using $\mathsf {BoolNet}$.

Identification of stable states in the resultant logical model resulted in three attractors consisting of one single attractor and two simple cycle attractors having two network states. The single attractor corresponds to the G$_{1}$ attractor and has a basin of 1,472 states, or approximately $71.88\%$ of initial states. The cycle attractors are composed of the following states: (1) {00001101110,  00000000001} and (2) {00000011010,  00000000011}, corresponding to a basin size of 370 states and 206 states, respectively. States of genes are encoded in the following order: Cln3, MBF, SBF, Cln1,2, Cdh1, Swi5, Cdc20,14, Clb5,6, Sic1, Clb1,2, and Mcm1. Except for the observed G$_{1}$ attractor, the presence of cycle attractors does not match the observations made by Li et al. [32]. We may consider the two cycle attractors as spurious limit cycles. In summary, the flexibility of factor graph formalism can allow us to implement certain biological processes and decisions that would otherwise be neglected by traditional Boolean approaches.

### Computation cost

This section shows how we performed simulation analysis using random Boolean networks of ten nodes to illustrate the computational cost of our proposed methodology. Since the factor graph representation of a network preserves the network complexity [30], the main computational cost of running the proposed methodology is the network update strategy using the proposed message-passing model.

**Fig. 7:**
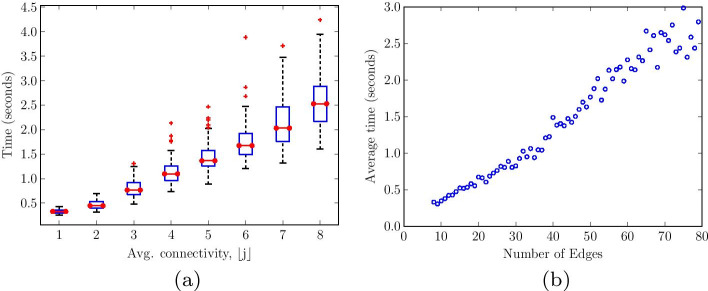
Time to evaluate the global attractors in random Boolean networks of 10 nodes with respect to: **a** average connectivity of control nodes, and **b** total number of edges in the network. The running cost depicts a linear performance with the graph connectivity

Figure 7 shows the performance of our proposed methodology in terms of the computational cost in searching the global attractors in a network. The search depicts a linear time computation with the average connectivity of control nodes or with the total number of edges in the network, as illustrated in Fig. 7a, b, respectively. This observation is consistent with findings in the literature regarding the computational complexity of message-passing algorithms in factor graphs where the computational cost grows linearly with the average degree of nodes times the number of nodes [17].

To describe an empirical estimation of the proposed model’s computational complexity, we assume that the entire network state space has been pre-computed and stored, which can be done offline. As such, searching the global attractors incurs ${\mathcal {O}}(2^{n})$, where *n* denotes the number of network nodes. According to our message-passing algorithm, the computation at the control nodes occur in parallel. Therefore, in each network update or iteration, a control node performs $k_{i}$ Boolean computations. Recall that $k_{i}$ corresponds to the number of edges between parent-child nodes. Given that each Boolean computation has a constant complexity of ${\mathcal {O}}(1)$, the cost of performing $k_{i}$ Boolean computations is bounded by ${\mathcal {O}}(k_{i})$. Moreover, the cost of computing the majority vote over $k_{i}$ values is ${\mathcal {O}}(k_{i})$. Thus, the total time complexity of a control node is ${\mathcal {O}}(k_{i})$.

On the other hand, a variable node in our proposed model simply sends out the value of its current state. This incurs a constant time complexity of ${\mathcal {O}}(1)$. Therefore, the overall time complexity of running our proposed model is ${\mathcal {O}}(k_{i})$ per iteration for each pair of variable and control nodes in searching the attractor of an initial state. Moreover, in message-passing algorithms, the number of iterations is always limited when the algorithm converges. Hence, for a constant number of iterations, the complexity of our model is proportional to the total number of edges in the graph, i.e., of order ${\mathcal {O}}(\sum _{i=1}^{n}k_{i})$. This is a linear time complexity and is consistent with the results in Fig. 7. For large biological networks, e.g., genome-wide regulatory networks, where the node connectivity is sparsely distributed, the complexity is linear in the number of nodes.

## Discussion

Identification of all attractors in a biological network is one of the key aspects in understanding the nature and dynamics of a biological system. In the literature, attractors have been found to fall into three groups, namely singletons, simple or limit cycles, and complex attractors [49]. For BNs of moderate size, i.e., networks with less than 20 nodes such as the illustrative Li model used in this work, our proposed model and methods can allow us to identify the attractors from the initial network states without the need for using a parallelized algorithm to reduce the computation time. However, as the network size increases beyond 20 nodes, the number of initial states to test grows exponentially. One can go around this limitation by specifying a subset of nodes in which all combinations are tested as noted in [49], or using a heuristic search starting from a number of predefined or randomly chosen states [21]. Similarly, other works in the literature also indicate that network reduction methods [54–56] can be employed to handle the analysis of large models [20].

Here, our proposed model and methods rely on simulations or enumerations of states to identify network global attractors. Thus, our model incurs a computational cost of evaluating state transitions online compared to some classical Boolean models such as GINsim and BoolNet that enumerate all state transition graphs or tables offline before identifying the network attractors. We note that the proposed approach may increase the computation cost, in particular when an extensive attractor search for a large network model is required. However, message passing can allow us to access and explore the dynamics of the interactions in a network after perturbations. Also, it provides a step towards understanding the impact of perturbations and how they propagate in the network. Furthermore, by employing network tools such as connectivity analysis and density evolution, we can gain insights for characterizing the resilience of biological networks to perturbations. In future work, we would implement a simulation approach that allows the control nodes to learn and compute the output states for a unique set of input values, and then use the learned model to perform simulations. This would reduce multiple computations of similar input values.

Based on the proposed model, we derived a density evolution equation to study the dynamics of error propagation in biological networks with random state disturbances. For instance, our DE analysis resulted in a necessary condition on the node degree distributions for biological systems to heal after an initial state perturbation. Our findings further revealed that low average connectivity may preclude the homeostatic stability of cellular systems since the number of attractors becomes high. Also, we note that our model further supports the conclusions made in [57], that simple Boolean function models can provide a means to reproduce and predict some biologically relevant dynamic features and network perturbation effects without full knowledge of biochemical kinetic parameters. However, these simplified models do not in any way render the precise dynamical models useless. Precise dynamical rules have a real advantage of modeling biological systems more accurately, albeit at an increased computational cost.

Despite their limitations and simple nature, Boolean networks have proven to be effective for qualitatively explaining the dynamics of biological systems. For instance, BN models have been found useful for the analysis of large-scale dynamic systems in which a detailed kinetic characterization is not feasible due to either limited knowledge or data restrictions. Though not covered here, gene over-expression can be implemented using our proposed methodology by fixing the state of a particular node in a network to a value of one.

## Conclusion

Computational models have been increasingly used to deduce and understand the nature of molecular interactions in biological systems and are widely accepted by the scientific community. Here, we have demonstrated that complex biological systems can be encoded into mathematical models. We explored a Boolean factor graph model representation of biological networks and applied a message-passing algorithm to study and analyze the behavior of genetic graphs as well as to predict the consequences of structural perturbations in biological networks. We verified the validity of our proposed model to characterize the dynamics of the yeast cell cycle and the consequences of gene deletion. For the simplified Li model sample network used, our Boolean factor graph model is able to capture the high-level dynamics of protein states, which is consistent with other published reports in the literature. Our findings imply that even in a larger cell-cycle network with multiple interactions and components performing similar functions, we can expect to infer fine details on how structural changes in a network affect its long-run dynamics. In addition, we have deduced that the yeast cell cycle is not only robust [32] but remains stable under diverse experimental conditions.

A possible future path would be to focus on deriving optimal interventions in genetic graphs based on a recursive equation model for both state and structural perturbations. Moreover, to adequately explain and obtain useful results in complex or large biological networks, it is imperative to extend our Boolean factor graph model to capture more meaningful biological behaviors such as temporal and modular.

## Supplementary Information

**Additional file 1**. Introduction to factor graphs and supplementary tables on gene deletion analysis.

## Data Availability

Processed data and proposed model implementation codes using MATLAB are freely available at https://github.com/kotiang54/bFGN_model. The full dataset is available from the Many Microbe Microarrays database (M$^{\mathrm {3D}}$).

## Abbreviations

DE: Density evolution
BN: Boolean network
KO: Knockout
DNA: Deoxyribonucleic acid
