# Supplementary material for: Boolean factor graph model for biological systems: the yeast cell-cycle network
Source: BMC Bioinformatics. 2021 Sep 17;22:442. doi: 10.1186/s12859-021-04361-8 (PMC8447535; doi:10.1186/s12859-021-04361-8)
Supplement: Supplementary file 1 — Additional file 1. Introduction to factor graphs and supplementary tables on gene deletion analysis. [file 12859_2021_4361_MOESM1_ESM.pdf]

Supplementary Materials

Boolean factor graph model for biological systems: The yeast cell-cycle network

Stephen Kotiang and Ali Eslami*

Department of Electrical Engineering and Computer Science, Wichita State University, Wichita, KS, USA.

1 Supplementary Notes

1.1 Factor Graphs

In general, we define a factor graph for variables $x_1, \cdots, x_n$ and functions $f_1, \cdots, f_K$, to be a bipartite graph on a set of nodes (known as variable nodes) corresponding to the variables, and a set of nodes (referred to as control nodes) corresponding to the functions [30]. Each control node depends on a subset of variable nodes, i.e., an edge exists between variable node x_i and control node f_k, if and only if x_i is an argument of f_k. The joint distribution over the variables is given by

$$p(x_1, \cdots, x_n) = \prod_{k=1}^{K} f_k\left(x_{C_k}\right), \tag{1}$$

where K is a discrete index set, C_k is the index set of variables that are connected to the function f_k, and x_{C_k} denotes this set of variables. Also, we refer to each control $f_k\left(x_{C_k}\right)$ in (1) as a local function. Figure S1 shows an example of a function, $p(x_1, x_2, x_3, x_4)$, on a bipartite graph that can be obtained as the product of $f_1(x_1, x_2)f_2(x_2, x_3, x_4)f_3(x_1, x_3)f_4(x_4)$.

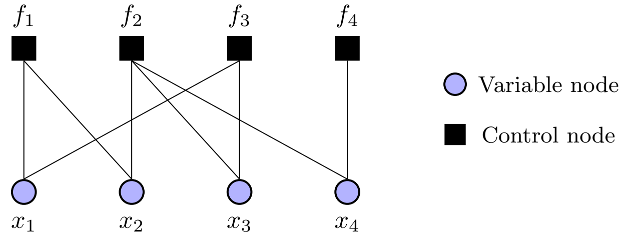

Figure S1: Factor graph for the product $f_1(x_1, x_2)f_2(x_2, x_3, x_4)f_3(x_1, x_3)f_4(x_4)$.

In the systems biology research area, factor graphs have been employed to refine gene regulatory network models with improved fit to experimental data and to characterize the steady-state behavior of Bayesian gene networks [37, 39]. Since biological networks are cyclic in nature, authors in [37] and [39] implemented a loopy belief-propagation algorithm as a message-passing inference tool. Also, in [36], the authors established a direct relationship between Boolean network models of gene regulatory networks and low-density parity-check codes decoding algorithms in a quest to study error correction in biology.

*Correspondence: ali.eslami@wichita.edu (Ali Eslami)

Using the formalism of BN models to represent genetic graphs, the nodes of this graph denote genes and a directed edge exists between two interacting genes. Thus, the expression level of one gene can be modeled using Boolean variables; gene i can either be 0 (repressed) or 1 (expressed) [1]. Having n genes, the global expression pattern can be described in terms of n-dimensional Boolean vector $\mathbf{x} = (x_1, \cdots, x_n) \in \{0,1\}^n$, where the gene-regulatory mechanisms are modeled as Boolean functions. An example of a gene network with $n = 3$ genes along with its bipartite form, is shown in Figure S2. Here, a control node represents a Boolean function. The expression level or state x_i of gene i is determined by a set of C_k expression levels x_{C_k}:

$$x_i = f_i\left(x_{i1}, x_{i2}, \cdots, x_{iC_k}\right) . \tag{2}$$

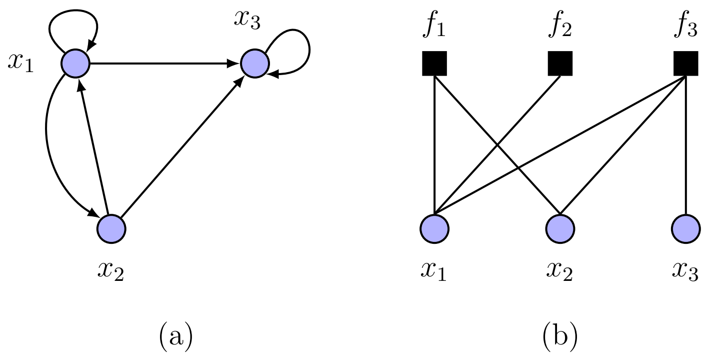

Figure S2: (a) Simple directed genetic graph with three nodes, and (b) equivalent undirected bipartite representation.

2 Supplementary Tables

Table S1: Temporal evolution of protein states for Li's model logical network [32]

Time	Genes											Phase
	Cln3	MBF	SBF	Cln1,2	Cdh1	Swi5	Cdc20,14	Clb5,6	Sic1	Clb1,2	Mcm1,SFF	
1	1	0	0	0	1	0	0	0	1	0	0	Start
2	0	1	1	0	1	0	0	0	1	0	0	G_1
3	0	1	1	1	1	0	0	0	1	0	0	G_1
4	0	1	1	1	0	0	0	0	0	0	0	G_1
5	0	1	1	1	0	0	0	1	0	0	0	S
6	0	1	1	1	0	0	0	1	0	1	1	G_2
7	0	0	0	1	0	0	1	1	0	1	1	M
8	0	0	0	0	0	1	1	0	0	1	1	M
9	0	0	0	0	0	1	1	0	1	1	1	M
10	0	0	0	0	0	1	1	0	1	0	1	M
11	0	0	0	0	1	1	1	0	1	0	0	M
12	0	0	0	0	1	1	0	0	1	0	0	G_1
13	0	0	0	0	1	0	0	0	1	0	0	Stationary G_1

Note: The right column indicates cell-cycle phases. Also, the number of time steps in each phase does not reflect its actual duration.

Table S2: Temporal evolution of protein states in Cdc20,14 gene deletion

Time	Genes											Phase
	Cln3	MBF	SBF	Cln1,2	Cdh1	Swi5	Cdc20,14	Clb5,6	Sic1	Clb1,2	Mcm1,SFF	
1	1	0	0	0	1	0	**0**	0	1	0	0	Start
2	0	1	1	0	1	0	**0**	0	1	0	0	G_1
3	0	1	1	1	1	0	**0**	0	1	0	0	G_1
4	0	1	1	1	0	0	**0**	0	0	0	0	G_1
5	0	1	1	1	0	0	**0**	1	0	0	0	S
6	0	1	1	1	0	0	**0**	1	0	1	1	G_2
7	0	0	0	1	0	0	**0**	1	0	1	1	M
8	0	0	0	0	0	0	**0**	1	0	1	1	M

Note: The cell cycle gets stuck in the M phase. Bold states in the sequence rows denote the state of the deleted node.

Table S3: Temporal evolution of protein states in Clb5,6 gene deletion

Time	Genes											Phase
	Cln3	MBF	SBF	Cln1,2	Cdh1	Swi5	Cdc20,14	Clb5,6	Sic1	Clb1,2	Mcm1,SFF	
1	1	0	0	0	1	0	0	**0**	1	0	0	Start
2	0	1	1	0	1	0	0	**0**	1	0	0	G_1
3	0	1	1	1	1	0	0	**0**	1	0	0	G_1
4	0	1	1	1	0	0	0	**0**	0	0	0	G_1

Note: The cell cycle arrests in the G_1 phase. The cell cannot initiate DNA synthesis (i.e., progress into the S phase). Bold states in the sequence rows denote the state of the deleted node.

Table S4: Temporal evolution of protein states in MBF gene deletion

Time	Genes											Phase
	Cln3	MBF	SBF	Cln1,2	Cdh1	Swi5	Cdc20,14	Clb5,6	Sic1	Clb1,2	Mcm1,SFF	
1	1	**0**	0	0	1	0	0	0	1	0	0	Start
2	0	**0**	1	0	1	0	0	0	1	0	0	G_1
3	0	**0**	1	1	1	0	0	0	1	0	0	G_1
4	0	**0**	1	1	0	0	0	0	0	0	0	G_1

Note: The cell executes the "Start" but arrests in the G_1 phase.

Table S5: Temporal evolution of protein states in SBF gene deletion

Time	Genes											Phase
	Cln3	MBF	SBF	Cln1,2	Cdh1	Swi5	Cdc20,14	Clb5,6	Sic1	Clb1,2	Mcm1,SFF	
1	1	0	**0**	0	1	0	0	0	1	0	0	Start
2	0	1	**0**	0	1	0	0	0	1	0	0	G_1

Note: The cell executes the "Start" but arrests in the G_1 phase.
